# Recent Advances in Simple Preparation of 3D Graphene Aerogels Based on 2D Graphene Materials

**DOI:** 10.3389/fchem.2022.815463

**Published:** 2022-01-26

**Authors:** Meichun Ding, Chenwei Li

**Affiliations:** ^1^ School of Chemistry and Pharmaceutical Engineering, Shandong First Medical University and Shandong Academy of Medical Sciences, Jinan, China; ^2^ Medical Science and Technology Innovation Center, Shandong First Medical University and Shandong Academy of Medical Sciences, Jinan, China

**Keywords:** graphene, aerogels, 3D porous materials, drying method, elasticity

## Abstract

Recently, 3D graphene aerogels (3GAs) with high electrical conductivity, excellent mechanical properties, and fast mass and electron transport have attracted increasing attention and shown wide applications (such as flexible electronics devices, sensors, absorbents, catalysis, energy storage devices, solar steam generation devices, and so on). The drying process becomes an important factor limiting the large-scale preparation of 3GAs. Therefore, how to simplify the preparation process plays an important role in the large-scale application of 3GAs. In this study, we summarize the recent progresses of 3GAs by different drying methods and focus on the effect of robust graphene network on the simple preparation of 3GAs. Besides, the design and synthesis strategies of 3GAs with robust graphene network structures have been systematically discussed. Finally, the emerging challenges and prospective for developing simple preparation and functionalization of 3GAs were outlined. It is expected that our study will lay a foundation for large-scale preparation and application of 3GAs and inspire more new ideas in this field.

## Introduction

The aerogel is a kind of three-dimensional porous material prepared from a precursor by a sol–gel process and a suitable drying method. The concept of “aerogel” was first presented by S. Kistler in the 1930s when he successfully obtained a porous SiO_2_ material of the same size as the original by using a supercritical drying method (Kistler, 1931a). Since then, inorganic aerogels (SiO_2_ aerogels, TiO_2_ aerogels, Al_2_O_3_ aerogels, and so on), organic aerogels (resorcinol–formaldehyde aerogels, melamine–formaldehyde aerogels, and so on), carbon-based aerogels, and so on have been widely used in many fields for their unique properties (low density, high porosity, high surface area, and low thermal conductivity). Since the 2010 Nobel Prize in Physics honored “the new 2D graphene material”, the atomic-thick graphene has attracted significant attention because of its unprecedented properties such as high electrical conductivity, excellent mechanical properties, high thermal conductivity, and high surface area (Novoselov et al., 2005). Thanks to the strong interactions between 2D graphene sheets, a tight face-to-face stack structure is formed. Therefore, 2D graphene materials are ideal building blocks for the synthesis of 3D graphene aerogels (3GAs) compared to 1D and 0D materials (Qiu et al., 2018). Until now, great efforts have been made to prepare a new family of 3GAs with extraordinary performances such as high electrical conductivity, excellent mechanical properties, and fast mass and electron transport, which conventional porous materials cannot offer (Lu et al., 2016; Huo et al., 2019; Li et al., 2019). 3GAs can be used in a range of applications including flexible electronics devices (Qiu et al., 2014), sensors (Qiu et al., 2016; Kong et al., 2021), absorbents (Bi et al., 2012), catalysis (Liang et al., 2018), energy storage devices (Jiang et al., 2017; Qu et al., 2020), and solar steam generation devices (Zhang et al., 2017; Yang et al., 2018; Huo et al., 2019; Li et al., 2021).

3GAs could be fabricated by using various methods such as the template-mediated assembly (Li et al., 2014), chemical vapor deposition (CVD) (Bi et al., 2015), 3D printing (Zhu et al., 2015), and self-assembly (Lu et al., 2016). Graphene oxide (GO) is a kind of functionalized graphene having an oxidation group such as an epoxy group and a hydroxyl group, which can be conveniently obtained by peeling off graphite oxide into a layered sheet (Zhao et al., 2015). Compared to other methods, the GO-based self-assembly method is the most promising method for large-scale preparation of 3GAs (Nardecchia et al., 2013). To form 3GAs, 2D GO sheets are assembled by dispersing GO in a solution. In the GO solution, the GO sheets are uniformly dispersed because of a balance between the electrostatic repulsion and van der Waals interaction (Cao et al., 2014). Gelation occurs by adding a crosslinking agent, sonicating the GO solution, changing the pH of the GO solution, and chemical or hydrothermal reduction to break the balance (Li and Shi, 2012). During the gelation process, the graphene sheets are chemically or physically assembled to form the graphene network structure. After gelation, graphene sheets are physically or chemically linked to form network structures; followed by the drying process, 3GAs are obtained (Nardecchia et al., 2013). In order to remove the water of graphene hydrogels, special drying techniques (such as freeze drying or supercritical drying) are often required due to the low solid content of graphene hydrogels (Ma and Chen, 2015). Due to the improvement of the preparation method, the cost of raw materials such as GO is continuously reduced (Kong et al., 2019). The drying process becomes an important factor limiting the large-scale application of graphene porous materials. Therefore, how to simplify the drying process plays an important role in the large-scale application of graphene porous materials. In this mini-review, the drying methods of 3GAs in recent years are summarized, and the influencing factors of mechanical performances of 3GAs are reviewed and prospected, in order to promote the large-scale application of 3GAs and inspire new ideas for the development of the simple preparation and functionalization of 3GAs**.**

### Drying Methods

#### Supercritical Drying Method

**FIGURE 1:**
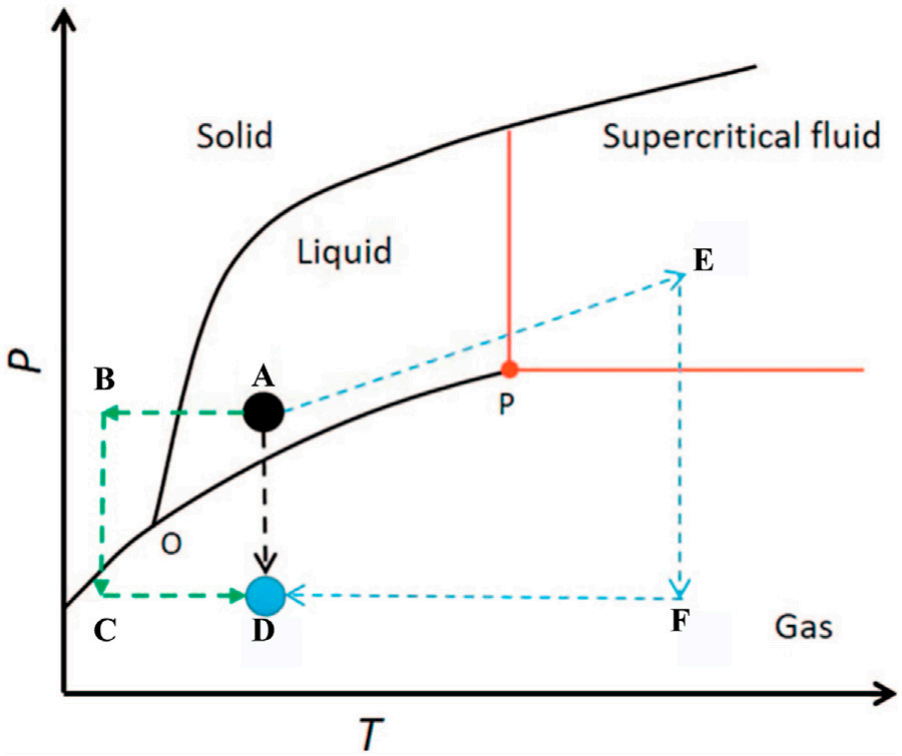
Single-phase phase diagram (Kistler, 1931b).

Due to the low solid content of graphene hydrogels, the graphene network collapses by the high capillary force of solvent evaporation when using the conventional drying methods (Li et al., 2016). In order to reduce the damage of the network structure by capillary force during the drying process, special drying methods (supercritical drying and freeze drying) are required. Supercritical drying is the most common method for preparing SiO_2_ aerogels (Zhang and Scherer, 2017). During the supercritical drying, the solvent (A spot in Figure 1) becomes a supercritical fluid (E spot in Figure 1) without a liquid–gas interface by adjusting the temperature and pressure, resulting into no capillary pressure in the network structures. When the temperature is kept above the critical temperature, the supercritical fluid can be removed (F spot in Figure 1) by depressurization to obtain aerogels (D spot in Figure 1) (Kistler, 1931b). The SiO_2_ aerogels dried by supercritical drying basically maintains the original network structure, and the amount of shrinkage is small (Zhang and Scherer, 2017). Compared with the SiO_2_ aerogel network composed of Si-O covalent bonds, the 3GA is constructed by graphene sheets through π–π interaction, and the graphene sheets are prone to slip during supercritical drying, resulting in a larger volume shrinkage and a larger density (Zhang et al., 2011; Sui et al., 2012; Huang et al., 2013a). Therefore, the supercritical drying method is not suitable for preparing low density and big-scale 3GAs. Moreover, the supercritical drying method involves the use of high pressure and a long-time process, resulting in high preparation costs.

### Freeze Drying Method

To eliminate capillary forces during the drying process, freeze drying is another option (Zhang and Scherer, 2017). The solvent in the wet gel is first replaced with water. Then, hydrogel (A spot in Figure 1) is first frozen (B spot in Figure 1) under low temperature. Finally, ice is sublimated into gas (C spot in Figure 1) under vacuum without going through the liquid state, thus keeping the graphene skeleton intact (D spot in Figure 1). For the SiO_2_ gel with a small pore size (tens of nanometer), the volume expansion of water after freezing may destroy the network structure, resulting in the decrease in porosity (as low as 80%) and the specific surface area (by half compared to supercritical drying) and cracking of the sample (Maleki, 2016). Due to the large pore sizes (several micrometers to several tens of micrometers), graphene hydrogels are more suitable for preparation using freeze-drying than SiO_2_ hydrogels. The 3GAs prepared by freeze drying have a lower density (as low as 0.16 mg cm^−3^), and the network structure can be controlled by ice crystals (Hu et al., 2013; Sun et al., 2013). However, the freezing of water may destroy and extrude the microstructure of the graphene network structure, resulting in the low specific surface area (Scherer, 1993). Moreover, freeze drying needs low temperature, high vacuum, and a long-time drying, resulting in high energy consumption and high preparation costs (Gallé, 2001).

### Vacuum Drying Method

The supercritical drying and freeze drying require the use of low vacuum, low temperature, or high pressure, resulting in the high cost of preparation of 3GAs, which will be challenging for large-scale production of 3GAs. Compared with supercritical drying and freeze drying, vacuum drying is a simple and time-saving process without requiring solvent exchange and expensive instruments for high pressure and/or temperature (Wang et al., 2013; Li et al., 2016). However, when the graphene gels are dried in a vacuum environment, the 3D network structures severely collapse, resulting in a sharp increase in the density. Therefore, vacuum drying is an appropriate way to fabricate the assembled structure of the 3D dense graphene network. Therefore, vacuum drying is a suitable method to fabricate the 3D dense graphene network assembly structure for high-volumetric/gravimetric energy density batteries (Li et al., 2018a).

### Air Drying Method

Air drying is a simple and low-cost technique for drying SiO_2_ gels under ambient pressure conditions and is more suitable for large-scale production of aerogels than supercritical drying and freeze drying (Maleki et al., 2015). However, according to the Young–Laplace equation (△P = 2γcosθ/r, where △P is the capillary pressure, γ is the surface tension of the solvent, θ is the contact angle of the air/fluid interface with the pore wall, and r is the pore size), the interface between liquid and air in a pore structure creates large capillary pressure during the air drying process due to the small pore size (tens of nanometer) (Smith et al., 1995). Because of the capillary pressure, the skeleton of the SiO_2_ gel gradually shrinks and cracks during the air-drying process. In order to reduce the capillary force, the solvent in the wet gel is first replaced with a solvent having a small surface tension (Zhang et al., 2011). Moreover, the skeleton of the SiO_2_ gel generally requires chemical treatment with a non-polar group to avoid further condensation of adjacent surface functional groups on the skeleton after compression by capillary stresses during the air-drying process (Maleki, 2016). For 3GAs, the pore structure is subjected to less capillary forces than SiO_2_ aerogels due to large pore sizes (several micrometers to several tens of micrometers). Therefore, 3GAs are more suitable for large-scale preparation using the air-drying method.

**FIGURE 2:**
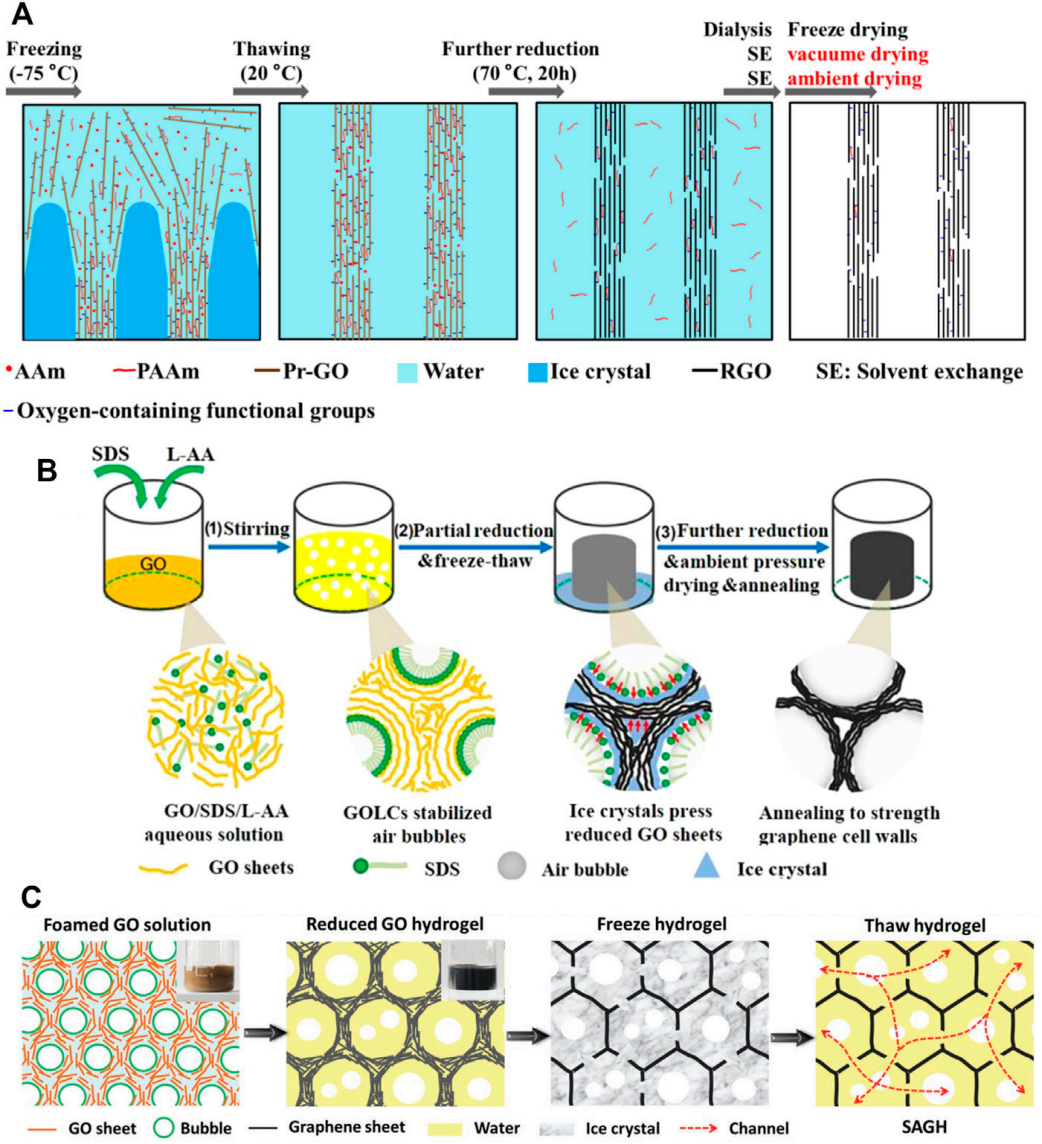
**(A)** Schematic model for the formation of the 3GA through *in situ* polymerization of Aam (Li et al., 2016). **(B)** Illustration of the preparation process of the 3GA by using GO liquid crystal-stabilized bubbles as a template (Zhang et al., 2019). **(C)** Schematic illustration of the air-dryable graphene hydrogel by using ice crystals and air bubbles as templates (Yang et al., 2019).

Recently, some researchers prepared the 3GAs by using the air-drying method. We fabricated robust and elastic (>80% strain) 3GAs through *in situ* polymerization of polyacrylamide to reinforce the graphene network (Figure 2A) (Li et al., 2016). No supercritical drying or freeze drying was required to prepare the 3GAs. The robust graphene network could resist the capillary force caused by solvent volatilization during vacuum drying and air drying and maintains low density (7.0–8.6 mg cm^−3^). Zhang et al. prepared the 3GAs by using GO liquid crystal-stabilized bubbles as a template (Figure 2B) (Zhang et al., 2019). The 3GAs with robust and uniform pore structures could be prepared using the air-drying method. The 3GAs can rapidly recover from the compression of 99% strain and maintain its original shape after 100,0000 compressive cycles at 70% strain. Yang et al. developed a method to prepare air-dryable graphene hydrogels by using ice crystals and air bubbles as templates (Figure 2C) (Yang et al., 2019). The superelastic 3GAs are obtained by a wet-press method based on the air-dryable graphene hydrogels. The 3GAs showed outstanding compressive stress (∼ 47 MPa) and superelasticity (can recover from the compression of 97% strain). Therefore, 3GAs that can be dried by air drying require robust graphene network structures to resist capillary forces. In general, these factors such as GO sheet sizes, interactions between graphene sheets, and graphene network structures are related to the mechanical performances of 3GAs. How to adjust these factors is critical to building robust graphene networks.

### Influencing Factors of the Mechanical Performances of 3GAs

#### GO Sheet Sizes

**FIGURE 3:**
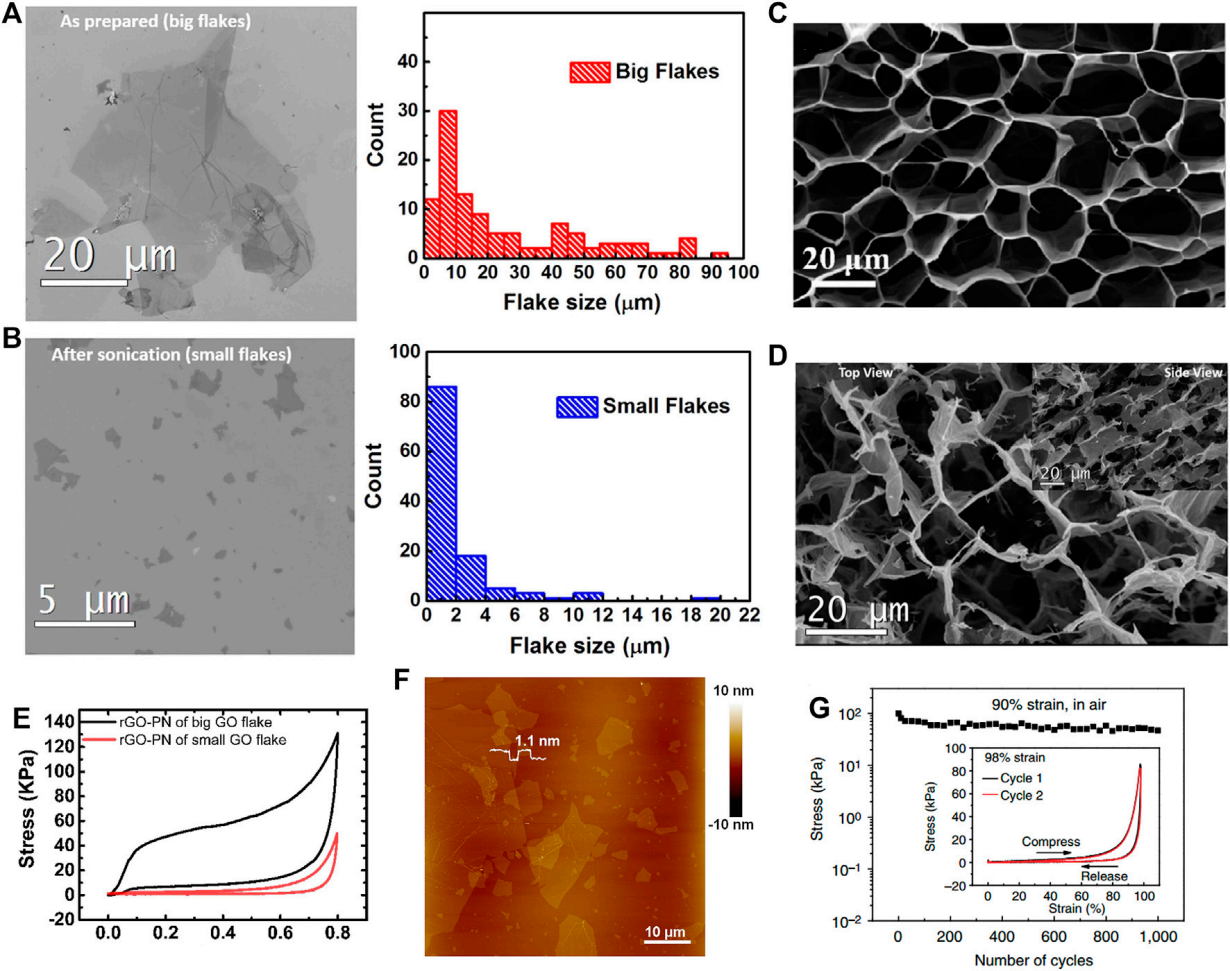
SEM images and size distributions of large **(A)** and small **(B)** GO sheets (Ni et al., 2015). SEM images of 3GAs prepared by large **(C)** and small **(D)** GO sheets (Ni et al., 2015). **(E)** Compressive stress–strain curves of 3GAs prepared by large and small GO sheets (Ni et al., 2015). **(F)** AFM image of GO sheets with a large size of 20–50 μm (Wu et al., 2015). **(G)** Compressive stress–strain curves of 3GA for 1,000 cycles in air. Inset: Compressive stress–strain curves of 3GA for two cycles under the compressive strain of 98% (Wu et al., 2015).

As the basic unit of a graphene network structure, the properties of the GO sheets affect the mechanical performance of the 3GAs. Based on the 3GAs prepared by using large GO sheets (∼ 20 μm) (Figure 3A) and small GO plates (∼ 2 μm) (Figure 3B), Ni et al. studied the effect of GO sheet sizes on the mechanical performance of 3GAs (Ni et al., 2015). The 3GAs prepared by large and small GO sheets showed honeycomb-like network structures (Figure 3C) and fragmented pore structures (Figure 3D), respectively. According to the results of compressive tests, 3GAs (small GO sheets) showed small compressive stress and elastic deformation during the compressive tests. The elasticity, compressive stress, and Young’s modulus of 3GAs produced by large GO sheets were significantly increased compared to the 3GAs produced by small GO sheets (Figure 3E) because large GO sheets can be assembled into a porous structure with a large π–π stacking area and fewer dislocations. Wu et al. also used the large GO sheets (20–50 μm) (Figure 3F) to prepare the 3GAs with superelasticity of up to 98% strain and excellent fatigue resistance (maintain structural integrity after 1,000 compression cycles) (Figure 3G) (Wu et al., 2015). Therefore, the use of large GO sheets is beneficial for the preparation of 3GAs with excellent mechanical properties.

### Interactions Between Graphene Sheets

**FIGURE 4:**
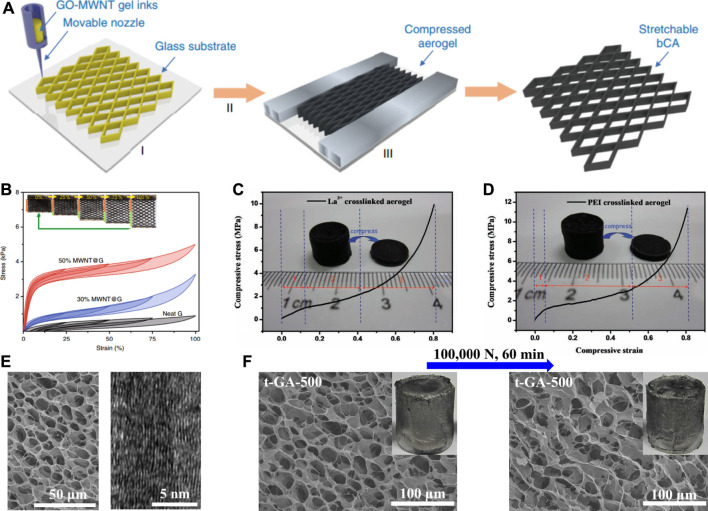
**(A)** Schematic illustration of the hierarchical synergistic assembly for fabrication of binary carbon porous material through the 3D printing technique (Guo et al., 2018). **(B)** Stress–strain curves of 3GAs with 25, 50, 75, and 100% tensile strains (Guo et al., 2018). Compressive stress–strain curves of the La^3+^- **(C)** and PEI- **(D)** crosslinked GO porous materials (Guo et al., 2018). **(E)** SEM image and HRTEM image of 3GAs (Li et al., 2018b). **(F)** SEM image and digital image of 3GAs before and after compression under 100, 000 N for 60 min (Li et al., 2018b).

Due to the graphene sheet stacks to construct the graphene network, it is an effective method to obtain robust 3GAs by enhancing the interactions between graphene sheets. Guo et al. fabricated binary carbon porous materials by the ink-printing solution of GO and multi-walled carbon nanotubes (MWNTs), followed by reduction and confined processes (Figure 4A) (Guo et al., 2018). Thanks to the hierarchical structures and synergistic effect of graphene and MWNTs, the binary carbon porous materials showed recoverable stretching elasticity (up to 100% strain) and excellent fatigue resistance (100,000 cycles) (Figure 4B). Huang et al. prepared two kinds of crosslinked GO porous materials by using La^3+^ and polyethylenimine (PEI) as crosslinkers, respectively (Huang et al., 2013b). The La^3+^- (55 mg cm^−3^) (Figure 4C) and PEI- (60 mg cm^−3^) (Figure 4D) crosslinked porous materials showed a Young’s modulus of 10 and 20 MPa and the compressive stress of 10 and 12 MPa, respectively. Due to the crosslinking of La^3+^ and PEI, the mechanical properties of crosslinked GO porous materials are much better than those without crosslinking. Based on the 3GAs prepared in our previous work (Li et al., 2016), we fabricated the 3GAs that could withstand extreme stress and strain through a simple thermal treatment (Li et al., 2018b). After the thermal treatment (400–1,000°C), most of oxygen-containing functional groups on the graphene sheets were eliminated, and π-π interaction between the graphene sheets were enhanced, forming an ordered stacked graphene sheets in cell walls (Figure 4E). The 3GA could recover its own shape and network structures after the extreme compression test (100, 000 N) of 60 min (Figure 4F). The 3GA exhibited the compressive stress of approximately 1,000 MPa and a strain of 99.8%, which are higher than those of other elastic porous materials.

### Graphene Network Structures

**FIGURE 5:**
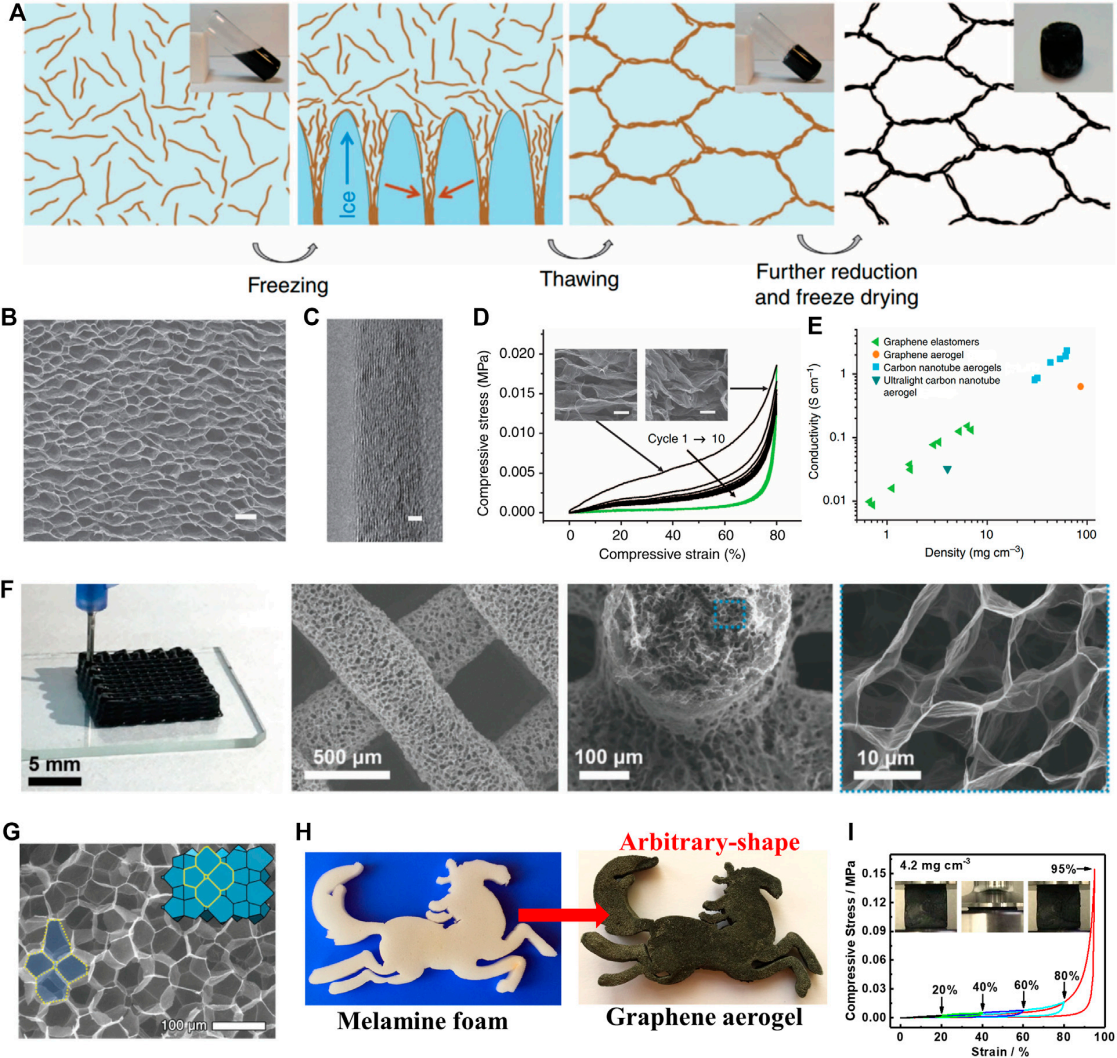
**(A)** Schematic illustrations for the fabrication of 3GA with ordered porous structure by using a two-step strategy (Qiu et al., 2012). SEM image **(B)** and HRTEM image **(C)** of 3GA (Qiu et al., 2012). **(D)** Compressive stress–strain curves of 10 cycles. Inset: SEM images of 3GA under compression during a cycle (Qiu et al., 2012). **(E)** The electrical conductivity of 3GAs was compared with those of other carbon-based porous materials as a function of density (Qiu et al., 2012). **(F)** Digital and SEM images of 3GAs prepared by the 3D printing technology (Peng et al., 2019). **(G)** SEM image of 3GA (Peng et al., 2019). **(H)** Fabrication of arbitrary-shaped 3GA by using melamine foam as the sacrificial skeleton (Li et al., 2018c). **(I)** Stress-strain curves of 3GA at a maximum compressive strain of 95% (Li et al., 2018c).

Because of the lack of effective control methods, GO sheets tend to assemble into a disordered graphene porous structure. These 3GAs with disordered network structures generally exhibit brittle and weak mechanical properties. How to construct ordered graphene porous structures is one of the key factors for preparing 3GAs with excellent mechanical properties. Recently, Qiu et al. used a simple method to fabricate elastic 3GAs with biomimetic hierarchical structures (Figure 5A) (Qiu et al., 2012). The ascorbic acid (reducing agent) was added to the GO solution, followed by a partial reduction (100°C, 30 min) and directional freezing (−78°C, 30 min). The partially reduced GO sheets were forced to align along the oriented ice crystals, resulting in the formation of ordered 3D network structures which were determined by ice templates. The weak hydrogel was strengthened due to the enhancement of π–π attraction during further reduction at 100 °C for 8 h. After freeze drying, 3GAs showed a honeycomb-like cell structures (Figure 5B) with cell walls composed of oriented graphene sheets (Figure 5C). The 3GA with a low density (5.1 mg cm^−3^) can withstand more than 50,000 times its weight under compression of 80% strain and retain its network structure intact, which was completely different from other 3GAs with random porous structures (Figure 5D). Meanwhile, the honeycomb-like cell structures and face-to-face stacked graphene sheets of the cell wall provide the excellent paths for electron transport, resulting in the high electrical property of 3GAs. The electrical conductivity of 3GAs is three times higher than that of the carbon-based porous material with a similar density (Figure 5E).

Researchers used other strategies to prepare robust 3GAs with ordered porous structures. Peng et al. used an ink-printing technique to construct ultralight and elastic 3GAs with biomimetic hierarchical structures (Figure 5F) (Peng et al., 2019). The macroscopic hollow scaffold and the microscopic honeycomb biomimetic-layered structure are realized by partially reducing the GO ink and combining the directional freezing process. The resulting 3GAs show exceptional resilience and a density of 8.5 mg cm^−3^. The 3GA can maintain its height of 90.1% after 10 cycles of 95% strain compression under the maximum pressure of 0.066 MPa. Yeo et al. used a strategy to prepare an ultralight and superelastic 3GAs with closed cellular structures (Figure 5G) (Yeo et al., 2018). First, spherical solid-shelled bubbles were prepared by controlled synthesis of functionalized GO sheets. Then, bubbles were assembled into a 3D network, resulting in the 3GAs with ordered rhombic dodecahedral structures through post-treatment. Benefiting from the ordered closed-cellular network for stress dissipation, the 3GA of 7.7 mg cm^−3^ exhibited the Young’s modulus of 0.3 MPa and retained the elasticity after the compression of 87% strain. Recently, we used melamine foam (MF) as the sacrificial skeleton to prepare the superelastic and durable 3GAs (Li et al., 2018c). The skeleton of MF effectively prevented serious accumulation of GO sheets, resulting in the arbitrary-shaped 3GAs with ordered network structures (Figure 5H). The 3GA with the low density (4.2 mg cm^−3^) showed the high elasticity with high compressive stress (0.556 MPa) and high compressive strain (95%) (Figure 5I).

## Discussion and Perspectives

The great potential of elastic and robust 3GAs in a variety of applications has drawn great attention although much process has been achieved on the simple preparation and functionalization of 3GAs. However, there are still some difficulties to overcome. First, most graphene gels have a fragile network structure and cannot withstand the capillary forces during air drying, resulting in structural collapse. Previous methods mainly connected graphene sheets *via* weak bonds (van der Waals force and ionic bond) to establish graphene networks. Therefore, it is necessary to develop an effective method to introduce more covalent bonds between graphene sheets in order to increase the stress between graphene sheets, forming the robust graphene network structure. Second, in order to obtain intact 3GAs by air drying, it is usually necessary to undergo a long-term solvent replacement and drying process, thereby increasing production costs. How to reduce the preparation cost of large-scale air drying? This requires the removal of time-consuming steps (such as replacement of water in the graphene hydrogel to a solvent having a small surface tension) and raising the temperature during air drying to increase the drying efficiency. Therefore, it is necessary to develop a new graphene hydrogel system that can withstand the damage of the graphene network by the capillary force caused by water evaporation at a high temperature. Third, in order to expand the application of 3GAs in electrocatalysis, energy storage, and electromagnetic shielding, it is also important to prepare 3GAs with mesoporous and microporous structures by using the air drying method. How to prepare the 3GAs with mesoporous and microporous structures and keep the mesoporous and microporous structure intact after air drying, which puts higher requirements on the design of the graphene hydrogel system. Therefore, continuous innovation and research and development are needed to further enhance the structure of the 3GA network, reduce the large-scale preparation cost of 3GAs, and expand the application range of 3GAs.
